# High-Frequency Spinal Cord Stimulation for the Treatment of Spasticity: A Preliminary Case Series

**DOI:** 10.3390/brainsci16010118

**Published:** 2026-01-22

**Authors:** Alessandro Izzo, Benedetta Burattini, Renata Martinelli, Quintino Giorgio D’Alessandris, Manuela D’Ercole, Maria Filomena Fuggetta, Nicola Montano

**Affiliations:** 1 Department of Neurosurgery, Fondazione Policlinico Universitario Agostino Gemelli IRCCS, 00168 Rome, Italy; alessandro.izzo@guest.policlinicogemelli.it (A.I.); quintinogiorgio.dalessandris@policlinicogemelli.it (Q.G.D.); manuela.dercole@policlinicogemelli.it (M.D.); mariafilomena.fuggetta@policlinicogemelli.it (M.F.F.); 2 Department of Neuroscience, Neurosurgery Section, Università Cattolica del Sacro Cuore, 00168 Rome, Italy; benedetta.burattini@gmail.com (B.B.); renata.martinelli01@icatt.it (R.M.)

**Keywords:** spasticity, spinal cord stimulation, high-frequency stimulation, neuromodulation, motor function

## Abstract

**Background:** Spasticity is a complex and multifactorial condition resulting from upper motor neuron injury. It manifests through muscle contractions, pain, limited range of motion, and clonus, which significantly impair daily activities and quality of life. High-frequency spinal cord stimulation (HF SCS) has shown optimal results in treating chronic neuropathic pain, but its potential role in spasticity remains underexplored. This study aimed to evaluate the efficacy of HF SCS in patients with spasticity. **Methods:** From April 2021 to July 2024, six patients with spasticity from various etiologies underwent SCS implantation at our institution. Clinical evaluations including the use of the Visual Analog Scale (VAS), Douleur Neuropathique 4 (DN4), and the Ashworth score, as well as ambulation ability and clonus episodes, were performed preoperatively and at a minimum of six months post-surgery. Subjective assessments of motor function, including coordination, movement efficiency, and postural transitions, were also recorded. **Results:** The mean age of patients was 50.12 ± 9.41 years, with follow-up averaging 24.32 ± 10.83 months. Statistically significant improvements were observed in VAS (*p* = 0.0412) and DN4 (*p* = 0.0422) scores, alongside a reduction in clonus episodes. All patients reported subjective improvements in coordination, movement efficiency, and postural transitions. Ambulation remained stable or improved in all cases. No perioperative complications or sensory/motor side effects were noted. **Conclusions:** HF SCS offers a promising approach to managing spasticity, with improvements in motor function, ambulation, and postural transitions. These findings support further investigation into HF SCS for spasticity, with multicenter trials needed to optimize treatment protocols and identify the most responsive patient populations.

## 1. Introduction

### 1.1. Definition, Pathophysiology and Clinical Manifestation of Spasticity

Spasticity is a complex and heterogeneous form of hypertonia resulting from injury to major descending pathways controlling spinal reflex excitability [1]. Although it has long been explained as a consequence of reduced supraspinal inhibitory input following an upper motor neuron lesion, modern models recognize the contribution of intrinsic spinal mechanisms, including enhanced motoneuron excitability, loss of presynaptic inhibition, and long-term plastic reorganization of reflex pathways. These processes collectively account for the clinical constellation of hypertonia, clonus, spasms, and abnormal reflex patterns observed in patients with chronic central nervous system (CNS) injury [2,3].

Common conditions in which spasticity represents one of the main and most disabling symptoms include multiple sclerosis, traumatic or ischemic brain and spinal cord injury, hereditary or genetic forms such as hereditary spastic paraplegia, and cerebral palsy. In these clinical scenarios, patients typically suffer from persistent muscle contractions, a reduction in the normal range of joint motion, pain, and involuntary rhythmic spasms known as clonus. These manifestations often interfere significantly with the performance of everyday activities and may severely impact overall quality of life.

Both supraspinal and spinal circuitry are believed to contribute to the pathogenesis and clinical expression of spasticity. Interruption of corticoreticulospinal pathways leads to impaired descending inhibition, while intrinsic spinal adaptations—such as increased motoneuron excitability, reduced presynaptic inhibition, and altered organization of segmental reflex circuits—further amplify motor output, leading to velocity-dependent hypertonia and abnormal reflex responses [3,4,5]. Modern models now emphasize, in fact, that the loss of supraspinal inhibition is only the initiating event. After an upper motor neuron lesion, segmental reflex circuits gradually become hyperexcitable through maladaptive spinal plasticity. This concept is supported by the typical sequence of early reflex depression (“spinal shock”) followed by a delayed return and exaggeration of reflexes, which cannot be explained by simple disinhibition alone. Processes such as collateral sprouting of afferent fibers, changes in synaptic organization, and denervation hypersensitivity are thought to contribute to the persistent increase in reflex excitability [2,3,6,7].

Importantly, different components of the upper motor neuron syndrome arise from distinct mechanisms: spasticity reflects a hyperactive tonic stretch reflex, clonus represents an oscillatory phasic stretch reflex, and flexor spasms originate from disinhibited nociceptive polysynaptic reflexes. These phenomena often coexist but are physiologically distinct. Secondary biomechanical changes resulting from prolonged muscle shortening further amplify resistance to movement and functional limitations [3].

### 1.2. Current Therapeutic Challenges

Given this complex and multifaceted pathophysiological background, the treatment of spasticity necessarily relies on the integration of multiple therapeutic approaches. The cornerstones of modern spasticity management include a structured rehabilitation process combined with pharmacological treatment, notably the use of botulinum toxin -primarily for focal spasticity- and baclofen, administered either orally or via intrathecal pumps [8,9]. These interventions aim to reduce disability, prevent long-term complications such as contractures and joint deformities, and ultimately improve patients’ quality of life and social participation. Nonetheless, baclofen therapy, particularly when delivered intrathecally, may present significant challenges, requiring careful dose management, regular pump refills, and close monitoring for multiple potential side effects [8,9].

### 1.3. Rationale of Spinal Cord Stimulation and Study Objective

In this complex therapeutic landscape, spinal cord stimulation (SCS) represents a promising and innovative approach, offering the advantage of being a fully reversible neuromodulation technique capable, at least in theory, of modulating motor pathways without the need for permanent lesions. Although the effects of SCS—mainly studied in nociceptive pathways—on motor circuits have been known for over 50 years, clinical application for spasticity has historically been limited. Several studies have reported potential clinical benefits in patients with spasticity treated with epidural SCS [10]; however, mixed and inconsistent results, the lack of well-conducted clinical trials, and the introduction of more established therapies such as botulinum toxin injections and intrathecal baclofen have curtailed its widespread adoption.

More recently, technological advancements in SCS therapy, including the development of fully magnetic resonance imaging (MRI)-compatible devices and the introduction of innovative waveforms and stimulation protocols, have renewed interest in SCS as a tool for neuromodulating motor pathways. These improvements may offer new rehabilitative opportunities for patients with spasticity, expanding the therapeutic landscape. In particular, high-frequency (HF) SCS delivered at 10,000 Hz (Nevro^®^ system, Nevro Corp., Redwood City, CA, USA) has emerged as a promising treatment paradigm for managing spasticity. Despite its theoretical advantages, clinical experience with HF SCS for this indication remains extremely limited. To date, only three reports have described the use of HF SCS in patients with spasticity, showing encouraging outcomes [11,12,13].

In parallel, a growing body of electrophysiological and translational research has demonstrated that both epidural and transcutaneous spinal stimulation can modulate key spinal mechanisms underlying spasticity. These approaches have been shown to reduce stretch reflex amplitude, enhance pre- and postsynaptic inhibition, and improve lower-limb motor function in individuals with spinal cord injury [13,14]. SCS may therefore represent a valid therapeutic option, particularly in patients with spasticity and partially preserved ambulation, in whom intrathecal baclofen may unpredictably interfere with functional motor strategies required for gait [15,16].

In this context, we present our preliminary experience, reporting clinical outcomes from a case series of six patients affected by both spasticity and neuropathic pain with partially preserved ambulation who underwent implantation with a high-frequency spinal cord stimulation device.

## 2. Materials and Methods

### 2.1. Study Design and Patient Selection

This study included six patients affected by both refractory spasticity of various etiologies and neuropathic pain who underwent implantation of a high-frequency spinal cord stimulation (SCS) system at our academic referral center between April 2021 and July 2024. All subjects were evaluated as part of our institutional clinical protocol for the management of refractory spasticity. The study was designed as a retrospective observational case series based on routine clinical practice. Given the exploratory nature of the study, treatment response was evaluated on a descriptive clinical basis rather than using predefined a priori criteria. Before device implantation, each patient underwent a comprehensive preoperative assessment, which included clinical examination and neuroimaging. In particular, all individuals received a dorso-lumbar spine magnetic resonance imaging (MRI) scan to exclude structural abnormalities that could interfere with lead placement.

### 2.2. Trial Phase and Definition of Clinically Meaningful Improvement

As part of the standard selection process, all candidates underwent a trial period of SCS lasting one month before permanent implantation. This trial phase consisted of percutaneous placement of an epidural lead connected to an external stimulator, during which stimulation parameters were progressively adjusted according to patient-reported effects on spasticity-related symptoms, pain, and motor performance. Clinically meaningful improvement during the trial phase was defined as a subjective patient-reported benefit associated with at least one of the following: reduction in clonus frequency or severity, improvement in neuropathic pain, or perceived improvement in motor control or functional performance. Patients who experienced such improvement were considered eligible for definitive internal pulse generator (IPG) implantation. During the study period, no patients with spasticity failed the trial phase. All patients experienced a perceived clinical benefit during the trial and independently decided to proceed with definitive implantation after shared clinical evaluation.

### 2.3. Surgical Procedure and Stimulation Parameters

In all cases, the epidural lead was positioned with the electrode tip located at the mid-thoracic level, corresponding to the anatomical “sweet spot” for lower-limb neuromodulation (approximately at the T8–T9 vertebral level) [17]. A high-frequency stimulation protocol (10 kHz) was used in all patients, delivered through a commercially available system (Nevro^®^). Stimulation parameters were programmed and subsequently adjusted during follow-up based on clinical response and tolerability.

### 2.4. Outcome Assessment

Outcomes were assessed at baseline (preoperatively) and at the latest available follow-up after definitive implantation. Concomitant antispastic and analgesic medications remained unchanged throughout the observation period. The evaluation included standardized scales widely used for the assessment of neuropathic pain and spasticity, namely the Visual Analog Scale (VAS), the Douleur Neuropathique 4 (DN4) questionnaire, and the Ashworth scale. VAS and DN4 scores were used exclusively to assess neuropathic pain. Both Ashworth scoring and pain assessments were performed by the same examiner at all time points to minimize inter-observer variability.

Additional functional parameters were also recorded, including ambulation ability, presence and frequency of clonus, and subjective motor performance. Clonus was clinically assessed by documenting its presence and the number of oscillations during elicitation, and changes were evaluated longitudinally within each patient. Ambulation ability was recorded as maximal walking distance with or without external support. Subjective motor performance was defined as the patient’s perceived improvement in activities such as changing position, carrying out daily living tasks, and executing coordinated movements more efficiently. These subjective measures were collected descriptively and were not interpreted as objective functional outcomes.

### 2.5. Rehabilitation Program

All patients continued their usual physiotherapy and rehabilitation programs throughout the postoperative period, following the same schedule adopted prior to SCS implantation. No structured modifications to rehabilitation protocols were introduced as part of the study.

### 2.6. Statistical Analysis

All data were analyzed descriptively and presented as mean ± standard deviation or median values when appropriate. For the comparison of continuous variables between preoperative and postoperative assessments, the Wilcoxon signed-rank test for paired samples was applied, given the small sample size and non-parametric distribution of the data. A *p*-value < 0.05 was considered indicative of statistical significance. We also calculated the Effect Size d_Cohen_ to evaluate mean differences. Statistical analyses were performed using StatView, version 5 (SAS Institute Inc., Cary, NC, USA).

## 3. Results

The clinical characteristics of the six patients included in this study are summarized in Table 1. Individual demographic and clinical data for each patient are reported in Supplementary Table S1. The mean age of the cohort was 50.12 ± 9.41 years, and the average follow-up (FU) duration was 24.32 ± 10.83 months. All patients completed the entire follow-up protocol, and no dropouts occurred during the observational period. At the most recent FU, we documented a statistically significant reduction in both pain intensity and neuropathic symptoms. Specifically, the Visual Analog Scale (VAS) score showed a significant improvement (*p* = 0.0412; Wilcoxon signed-rank test), indicating meaningful relief from chronic pain. Similarly, the Douleur Neuropathique 4 (DN4) questionnaire score decreased significantly (*p* = 0.0422), supporting a reduction in neuropathic sensory disturbances. A mild decrease in muscle tone, as assessed by the Ashworth scale, was also observed; however, this change did not reach statistical significance (Table 2). Clonus was present in all patients before surgery. At the latest FU, four out of six individuals exhibited a clear clinical improvement, with complete disappearance of clonus in three cases. The remaining two patients demonstrated stability without worsening. Quantitatively, no significant difference was found in number of clonus oscillations at FU compared with the preoperative (*p* = 0.065; Wilcoxon signed-rank test), even though an overall reduction was observed.

Ambulatory function was also evaluated. In four patients, gait remained stable compared to baseline. Remarkably, two individuals experienced substantial functional gains: one patient increased autonomous walking distance from 30 to 200 m, while another improved from 10 to 100 m without requiring external support (Table 2). Beyond measurable parameters, all patients reported subjective improvements in motor performance, describing increased movement fluidity, better coordination, and greater ease in performing postural transitions such as standing up, sitting, or changing position. These self-reported functional enhancements are consistent with the objective reductions in spasticity-related phenomena.

Large paired effect sizes (Effect Size d_Cohen_) were observed for VAS, DN4, and clonus reduction, with a moderate-to-large effect for Ashworth scores. Ambulation outcomes were highly variable across patients, resulting in a smaller overall effect size (see Table 2).

Importantly, no perioperative complications occurred, and no patient experienced motor or sensory adverse events attributable to SCS during the entire follow-up period. Device-related issues were absent, and stimulation remained well tolerated in all cases. This confirms the overall safety, feasibility, and acceptability of the procedure in this patient population.

## 4. Discussion

Although several therapeutic strategies are currently available for the management of spasticity, their efficacy and applicability vary widely across patients, especially in those with complex or mixed motor presentations.

For many decades, ITB has represented the cornerstone of treatment for generalized spasticity. Nonetheless, this therapy can be difficult to manage in some cases [8,9] and is not always suitable for individuals with partially preserved ambulation, as its effects may unpredictably interfere with functional motor strategies required for gait [15,16,18].

While ITB is an effective treatment for spasticity, its functional benefits may be limited in many ambulatory patients. This is due to its narrow therapeutic window, where small adjustments in dosage can result in either insufficient effectiveness or excessive weakness. This is particularly concerning for patients who rely on residual hypertonia for gait stability [18,19].

This therapeutic gap highlights the need for alternative neuromodulatory approaches capable of reducing spasticity without impairing gait-supporting strength.

The role of SCS in treating spasticity was only investigated in the past when tonic stimulation (a kind of stimulation paradigm requiring the evocation of leg paresthesia) was available, leading to mixed results, but an overall symptomatic improvement in most cases was also reported [20]. HF SCS has long been used in chronic pain management [21], and its role in treating spasticity has recently been explored in 4 patients [11,12,13]. Thus, to the best of our knowledge, this paper reports on the largest cohort of spasticity patients submitted to HF SCS. Compared with previous reports, the present study contributes novel clinical insights by focusing on patients with partially preserved ambulation, systematically reporting clonus as an outcome measure, and providing the largest case series to date specifically evaluating high-frequency SCS for spasticity.

Recent preclinical evidence also supports the potential role of SCS in modulating spasticity: in a contusive SCI rat model, high-frequency epidural stimulation delivered at adequate intensities significantly reduced both spasticity and neuropathic pain, an effect associated with decreased microglial activation in dorsal and ventral spinal horns. These findings provide mechanistic support for the use of SCS as a neuromodulatory strategy in disorders characterized by abnormal spinal excitability [22].

In our case series, we documented a consistent improvement in neuropathic symptoms across all treated patients. Notably, each patient reported enhanced coordination, reduced fatigability, and smoother postural transitions following HF SCS. While these findings are directly supported by the observed clinical data, mechanistic interpretations regarding the effects of HF SCS on spinal excitability should be regarded as hypothesis-generating and interpreted with caution, given the exploratory nature of the study.

In addition, we observed a reduction in clonus episodes in every case, including those involving patients with MS. In such patients, the efficacy of SCS in controlling neuropathic pain and alleviating urinary symptoms has also been confirmed by a recent systematic review [23].

Although some studies have reported partial relief of motor symptoms in patients with MS, the supporting evidence remains limited and inconclusive. This is largely due to the intrinsic heterogeneity of the disease, the variability in patient selection, and the inconsistency of stimulation protocols across studies. Nonetheless, clonus reduction may significantly facilitate motor function and represents a valuable therapeutic target, particularly when integrated within structured rehabilitation programs.

In our cohort, clonus improvement emerged as one of the most consistent findings and may constitute a particularly sensitive marker of segmental spinal modulation. Clonus is generated by self-sustained oscillations within Ia afferent–α-motoneuron loops -driven in part by increased persistent inward currents (PICs) and reduced supraspinal inhibition- and is therefore highly dependent on segmental spinal excitability. This likely explains why clonus responded more readily to neuromodulation than Ashworth scores, which are strongly influenced by non-neural factors such as soft-tissue stiffness.

High-frequency SCS may attenuate these Ia-driven oscillations by modulating dorsal horn processing and enhancing inhibitory control over reflex pathways. The consistent improvement observed across our patients, including those with supraspinal lesions, supports the notion that clonus may serve as an early and sensitive clinical indicator of neuromodulatory efficacy [24,25].

Although SCS modulates both spinal reflex circuits and descending supraspinal pathways, its clinical effectiveness appears to be maximized when combined synergistically with intensive rehabilitation protocols, enhancing both functional recovery and overall patient outcomes [26].

In this context, SCS may act as a neuromodulatory primer, temporarily restoring a more physiological excitability state of spinal circuits and thereby enhancing responsiveness to rehabilitative inputs. This effect highlights the importance of a multidisciplinary approach, in which neuromodulation is integrated with targeted physiotherapy, gait training, and functional strengthening to maximize functional recovery.

A stable or even improved ambulation was another relevant finding in our series, with two patients (one affected by thoracic myelopathy and the other by hereditary spastic paraplegia) showing a dramatic enhancement in walking ability. The wide variability observed in ambulation outcomes is not unexpected and likely reflects the marked heterogeneity of the underlying neurological conditions, baseline functional status, and disease progression trajectories within this cohort.

These clinical outcomes support the potential utility of HF SCS in managing spasticity among patients who retain some degree of ambulation. The benefits appeared particularly evident in individuals with spasticity of spinal origin, compared to those with a cerebral etiology, where functional improvements were more limited [10,12].

This discrepancy may reflect fundamental differences in the underlying pathophysiology: spinal spasticity is largely mediated by segmental circuit hyperexcitability and reduced inhibitory control at the spinal level, mechanisms directly targeted by epidural neuromodulation. Conversely, spasticity arising from cerebral lesions, such as stroke or traumatic brain injury, often involves more complex maladaptive changes within supraspinal networks, including corticoreticulospinal reorganization and altered motor cortex excitability. These multifactorial supraspinal contributions may reduce the responsiveness to spinal-level stimulation and help explain the comparatively attenuated functional gains observed in patients with cortical or subcortical involvement.

The absence of complications in our cohort aligns with the well-established safety profile of spinal cord stimulation. Large multicenter studies on 10 kHz SCS have reported low rates of device-related adverse events and excellent long-term tolerability, comparable or superior to conventional stimulation. Although these trials focused on pain, they confirm that high-frequency stimulation can be delivered chronically without negative effects on motor or sensory function, supporting its feasibility in patients with spasticity [27,28].

### Limitations

This study has inherent limitations. First, it is a retrospective, uncontrolled case series including a very small number of patients with heterogeneous neurological conditions (multiple sclerosis, hereditary spastic paraplegia, and thoracic myelopathy), which differ in pathophysiology, disease progression, and potential responsiveness to neuromodulation. This heterogeneity may have influenced the observed motor and ambulation outcomes. Second, the lack of a control group limits causal inference regarding treatment efficacy. Moreover, objective quantitative motor outcome measures were not available, and functional assessments were mainly clinical; in progressive neurological disorders, disease evolution itself may further confound motor outcome interpretation. Finally, given the extremely limited sample size, statistical analyses should be interpreted with caution and considered primarily descriptive. Larger, prospective, and more homogeneous studies will be essential to determine which patient subgroups benefit most from high-frequency SCS and to confirm the consistency of the clinical effects observed in this preliminary series.

## 5. Conclusions

In conclusion, spasticity remains a complex condition requiring individualized, multidisciplinary management. In this preliminary case series, HF SCS appears to be a feasible neuromodulation option in selected patients with partially preserved ambulation, with potential benefits in neuropathic pain control, clonus reduction, and functional stability. Future prospective studies with larger and more homogeneous cohorts will be essential to refine patient selection criteria, optimize stimulation parameters, and better define the role of HF SCS within integrated rehabilitation programs.

## Figures and Tables

**Table 1:** Demographic and baseline clinical characteristics of the study cohort. Data are reported as mean ± standard deviation. MS: multiple sclerosis.

Number of patients	6
Sex (M/F)	3/3
Mean age (years)	50.12 ± 9.41
Diagnosis	(1) Relapsing-remitting MS (2) hereditary spastic paraplegia (3) thoracic myelopathy
Disease duration, years	21 ± 18.46
Follow-up (months)	24.32 ± 10.83

**Table 2:** Clinical outcomes at baseline and follow-up after high-frequency spinal cord stimulation. Values are reported as mean ± SD (median). Statistical comparisons were performed using the Wilcoxon signed-rank test for paired samples.

Parameter	Preoperative	Follow-Up	*p*-Value	Effect Size d_Cohen_
VAS	6.16 ± 2.63 (median 7)	3.16 ± 2.71 (median 2)	0.041	1.15
DN4	6.50 ± 3.25 (median 8)	1.83 ± 1.72 (median 2)	0.042	1.71
Ashworth scale	2.66 ± 0.51 (median 3)	2.16 ± 0.75 (median 2)	0.083	0.91
Number of clonuses	6.33 ± 3.83 (median 6)	1.83 ± 2.40 (median 1)	0.065	1.01
Ambulation ability (meters)	21.66 ± 9.83 (median 25)	65.00 ± 73.41 (median 30)	0.179	0.6

Legend: Visual Analog Scale (VAS); Douleur Neuropathique 4 (DN4). *p* < 0.05 significant. Cohen’s d: effect size (0.2 small, 0.5 moderate, ≥0.8 large).

## Data Availability

The data presented in this study are available on request from the corresponding author due to privacy restrictions.

## Supplementary Materials

The following supporting information can be downloaded at: https://www.mdpi.com/article/10.3390/brainsci16010118/s1, Table S1: Individual demographic and clinical characteristics of the study cohort.
