# Supplementary material for: High-Frequency Spinal Cord Stimulation for the Treatment of Spasticity: A Preliminary Case Series
Source: Brain Sci. 2026 Jan 22;16(1):118. doi: 10.3390/brainsci16010118 (PMC12838596; doi:10.3390/brainsci16010118)
Supplement: Supplementary file 1 [file brainsci-16-00118-s001.zip › brainsci-4069206-supplementary.pdf]

Supplementary Table S1

Table S1. Individual demographic and clinical characteristics of the study cohort

Case	Age	diagnosis	Disease duration (years)	VAS pre-op	VAS FU	Ashwort pre-op	Ashwort FU	DN4 pre-op	DN4 FU	Clonus pre-op (Number)	Clonus FU (Number)	Ambulation pre-op (meters)	Ambulation FU (meters)	FU (months)
1	43	MS	10	8	2	2	1	8	1	10	3	21	21	36
2	61	MS	14	6	6	3	3	5	5	11	0	10	100	30
3	52	hereditary spastic paraplegia	52	1	0	3	2	3	0	7	0	9	9	16
4	55	thoracic myelopathy	5	8	7	3	3	6	1	6	6	30	200	34
5	35	hereditary spastic paraplegia	35	7	2	3	2	8	2	2	0	28	28	18
6	53	MS	10	7	2	2	2	9	2	2	2	32	32	12

Legend: MS: multiple sclerosis; VAS: Visual Analog Scale for neuropathic pain; DN4: Douleur Neuropathique 4 questionnaire; FU: follow-up
